# Amenorrhea, fertility preservation, and counseling among young women treated with anthracyclines and taxanes for early-stage breast cancer, a retrospective study

**DOI:** 10.1097/MD.0000000000019566

**Published:** 2020-03-13

**Authors:** Hikmat N. Abdel-Razeq, Razan A. Mansour, Khawla S. Ammar, Rashid H. Abdel-Razeq, Hadil Y. Zureigat, Lina M. Yousef, Omar A. Shahin

**Affiliations:** a Department of Medicine, King Hussein Cancer Center; b School of Medicine, University of Jordan; c Office of Research and Scientific Affairs, King Hussein Cancer Center, Amman, Jordan; d School of Medicine, Hashemite University, Zarka, Jordan; e Department of Nursing, King Hussein Cancer Center, Amman, Jordan.

**Keywords:** breast cancer, chemotherapy, gonadotropin-releasing hormone agonists, pregnancy, premature ovarian failure, premenopausal

## Abstract

Chemotherapy may cause ovarian toxicity and infertility. Cancer patients are usually overwhelmed, and focus exclusively on cancer diagnosis and may not pay attention to fertility-related issues. In this paper we look at the rate of amenorrhea and fertility counseling among such young patients.

Premenopausal women with early-stage breast cancer treated with adjuvant or neoadjuvant chemotherapy were recruited. Amenorrhea was defined as absence of menstruation for ≥12 months after the completion of chemotherapy.

A total of 94 patients met the eligibility criteria and were included in this analysis. Median age at diagnosis was 35.7 (range, 22–44) years. Seventy-nine (85.9%) respondents were counseled about amenorrhea and 37 (40.2%) were considering having children. Long-term amenorrhea was reported by 51 (54.3%) patients. The addition of taxanes to anthracyclines, in 2 different regimens, increased the risk of amenorrhea to 69.2% and 66.7% compared to 38.9% with anthracycline-alone, *P* < .0001. Longer duration of chemotherapy (≥24 weeks) might also be associated with higher rate of amenorrhea (67.7%) compared to 43.4% in those who had shorter duration (<24 weeks), *P* = .031.

The addition of taxanes to anthracycline-based chemotherapy increased the risk of amenorrhea. However, shorter duration of chemotherapy, even with taxanes, may lower such risk. Our study highlights the importance of fertility counseling to improve fertility preservation rates. Given the importance of taxanes, shorter regimens are associated with lower amenorrhea rates and should be preferred over longer ones.

## 1 Introduction

Breast cancer continues to be the most common cancer among females worldwide^[1,2]^ and so in Jordan.^[3]^ Of the more than 6000 new cancer cases diagnosed annually among Jordanians, 20% are breast cancer and almost half of them are premenopausal at time of diagnosis. Additionally, more than 15.0%, compared to less than 7.0% in western societies,^[4]^ are below the age of 40 years at time of breast cancer diagnosis.^[3]^

The value of adjuvant chemotherapy in breast cancer, especially in younger patients, is undebatable. Published studies had shown that adjuvant chemotherapy prolonged both disease-free and overall survivals.^[5]^ However, it is also known that chemotherapy may lead to premature ovarian failure (POF), amenorrhea, and impaired fertility in this young age group. Early menopause may also result in higher risk of osteoporosis,^[6]^ cardiovascular diseases,^[7]^ genitourinary dysfunctions, vasomotor symptoms like hot flushes,^[8]^ and psychological disturbances.^[9]^

Previous published studies had reported variable rates of POF with cytotoxic chemotherapy. Much of the published literature focused on regimens incorporated anthracyclines and cyclophosphamide like adriamycin and cyclophosphamide (AC) and 5-flurouracil, epirubicin and cyclophosphamide (FEC) or the old cyclophosphamide, methotrexate and 5-fluorouracil (CMF) regimen. In 1 study, the use of either CMF or cyclophosphamide, epirubicin and 5-fluorouracil, alone or with endocrine therapy in young women with breast cancer, increased the risk of premature menopause from less than 5% to more than 40%.^[10]^

Very few studies addressed this issue among breast cancer patients treated with taxanes which is incorporated with almost all adjuvant or neoadjuvant breast cancer chemotherapy regimens.^[11,12]^ In 1 study, researchers from the Memorial Sloan Kettering Cancer Center reported their experience on 166 premenopausal women aged 40 years or younger (median age: 36 years), all were treated with adjuvant anthracycline and taxane-based chemotherapy for early-stage breast cancer. Twenty-five (15%) patients developed POF and long-term amenorrhea. Women who experienced amenorrhea were found to be significantly older than those who did not (*P* < .01).^[13]^

Despite its significant impact on their quality of life, counseling about POF and infertility associated with chemotherapy is not routinely practiced. A recent study from Germany that recruited 1191 young women with locoregional primary breast cancer, treated between 2006 and 2014 looked at patient-reported outcomes on issues that included fertility concerns. The mean age at diagnosis was 40 years. Only half of all patients had been informed about the risk of infertility following chemotherapy. However, counseling at a specialized fertility center was offered to only 13% and only 2% underwent fertility preservation procedures.^[14]^ Given this low compliance rate, we put together a bundle of regulations to enhance our counseling and fertility preservation rates. Fertility counseling, both for males and females, was a key performance quality indicator; data on which is regularly collected and reported by our quality office. Additionally, fertility preservation is addressed while discussing the medical management of our young breast cancer patients during our twice-weekly multidisciplinary clinic breast cancer meetings. A fertility-preservation clinic run by an experience gynecologist was also established.

In this paper we look at the rate of amenorrhea and fertility counseling among young females diagnosed with breast cancer and treated with anthracycline with or without taxanes in a developing country like ours.

## 2 Methods

Premenopausal women with early-stage breast carcinoma aged 44 years or younger treated and followed at our center for at least 12 months after the completion of all chemotherapy were identified. Eligible patients were required to have invasive breast carcinoma treated with anthracycline and/or taxane-based adjuvant chemotherapy. Patients progressed during the first 12 months were excluded. Additionally, patients underwent surgical or medical ovarian ablation using gonadotropin-releasing hormone (GnRH) agonists (n = 79 were also excluded. Patients could have received endocrine therapy as indicated by the hormone receptor status of their tumors.

Long-term amenorrhea was defined as absence of menstruation for 12 months after the completion of all chemotherapy. Follow-up evaluations were performed at 3-month intervals during the first year and every 4 months during the second and third year then every 6 months intervals thereafter. Information regarding menstrual status was obtained from patients during their follow up and/or a review of the medical records. A self-reported questionnaire was given for patients enrolled. Patients who agreed to participate signed a consent form consistent with our institutional review board guidelines.

The 2 primary study outcomes were the rate of fertility counseling, provided by the treating physician or medical team before initiation of chemotherapy, and the rate of POF. These outcomes were obtained from patients’ study questionnaires and medical record review. In addition to a series of sociodemographic questions, patients were required to respond to a group of questions related to menstrual cycle before and after chemotherapy, it also contains questions related to marital status at time of cancer diagnosis, prior marriage, prior kids and number, and their desire to have future ones.

Patients were also asked a series of “yes” or “no” questions regarding whether the treating medical team members, had discussed the risk of POF and potential infertility, and whether recommendations for ova preservation were made.

Study was reviewed by the institutional ethics committee and consents were waived.

## 3 Results

Between January 2014 and December 2017, a total of 94 patients met the eligibility criteria and were included in this analysis. The median age at diagnosis was 35.7 (range, 22–44) years. Majority (67, 71.3%) of the patients were married and most had a relatively low-income. Patient characteristics and pathological features of the tumor are detailed in Table 1. As illustrated, 56 (59.6%) patients had node-positive tumors while almost a third had human epidermal growth factor receptor-2-positive disease.

**Table 1:**
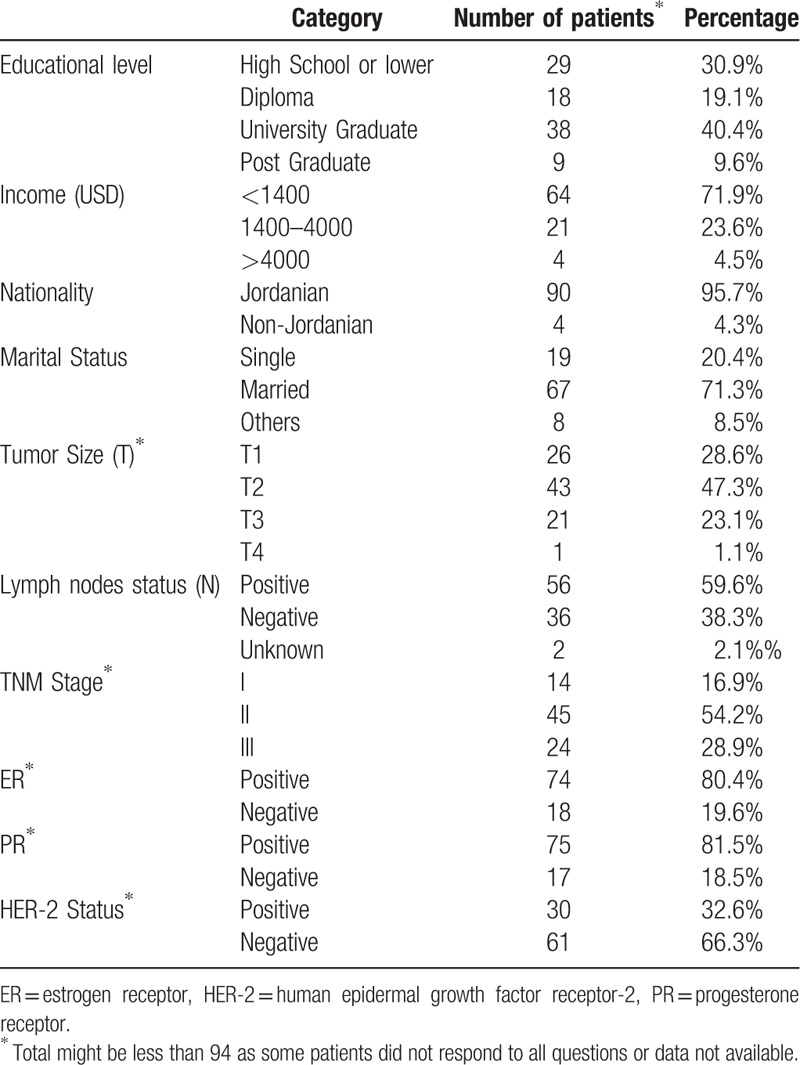
Patients characteristics.

All patients had regular menses at time of diagnosis and before initiation of adjuvant chemotherapy. Treatment schedule and dosing are detailed in Table 2. Briefly, 4 cycles of AC (doxorubicin at a dose of 60 mg/m^2^ plus cyclophosphamide at a dose of 600 mg/m^2^) delivered every 3-week. Docetaxel was given at 100 mg/m^2^ every 3 weeks while paclitaxel was given at a dose of 85 mg/m^2^ weekly for 12 weeks. Additionally, FEC (5-flurouracil at 500 mg/m^2^, epirubicin at 50 mg/m^2^, and cyclophosphamide at 500 mg/m^2^) was given every 3 weeks.

**Table 2:**
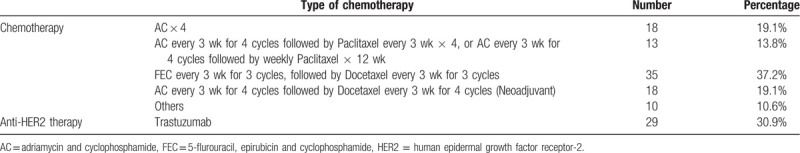
Chemotherapy and anti-HER2 treatment.

In the adjuvant setting, low-risk, node-negative patients were treated with AC regimen for 4 cycles, while high-risk node-negative patients and all node-positive patients were given anthracycline-based chemotherapy (AC for 4 cycles or FEC for 3 cycles) followed by a taxane (paclitaxel weekly for 12 weeks or docetaxel every 3 weeks for 3 cycles). In the neoadjuvant setting, all patients were treated on a unified program; 4 cycles of AC followed by 4 more cycles of docetaxel, all delivered every 3 weeks.

A total of 84 (89.4%) patients received anthracycline-based chemotherapy while 66 (70.2%) patients received additional taxanes. Seventy-eight (83.0%) were on hormonal therapy with tamoxifen, too.

Seventy-nine (85.9%) respondents were counseled by their oncologists about amenorrhea as a complication of chemotherapy and 37 (40.2%) were considering having children post treatment and 43 (46.7%) stated that they would use the service.

Fifty-one (54.3%) patients developed long-term amenorrhea. Patients who were treated with anthracycline based chemotherapy regimen in the form of AC without taxanes (n = 18) had significantly lower rate of amenorrhea (38.9%) compared to a rate of 69.2% among those who received an additional 12 weeks of weekly paclitaxel (n = 13), and 66.7% among those (n = 18) who received an additional 4 cycles of docetaxel every 3 weeks, *P* < .0001. Amenorrhea was reported by 16 (45.7%) of 35 patients who received 3 cycles of FEC followed by 3 more cycles of docetaxel, all were given at 3-week intervals. Combined together, the rate of amenorrhea was significantly higher (56.1%) with taxanes-containing regimens compared to anthracycline-alone regimen (38.9%), *P* = .024 (Table 3).

**Table 3:**
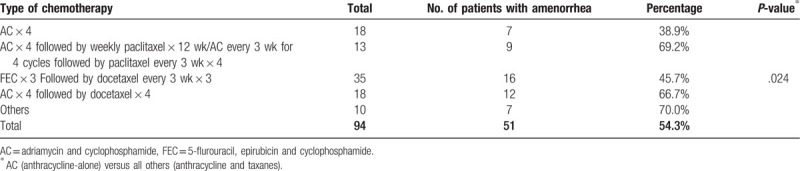
Rate of amenorrhea according to chemotherapy regimen.

A total of 31 patients had chemotherapy for more than 24 weeks, 21 (67.7%) had amenorrhea compared to 23 (43.4%) of 53 patients who had a shorter duration of chemotherapy, *P* = .031 (Table 4). Only 7 (7.4%) patients where 30 years or younger at time of chemotherapy administration; 2 (28.6%) of them had amenorrhea compared to 49 (58.3%) of 84 patients above the age of 30 years, *P* = .127

**Table 4:**
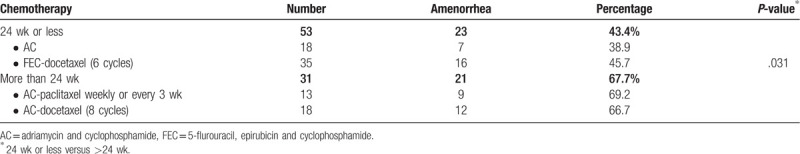
Rate of amenorrhea according to duration of chemotherapy.

## 4 Discussion

Population of Jordan is younger one; almost 38% of Jordanian females are younger than the age of 40.^[14]^ This, among other factors, might explain the lower incidence of breast cancer in the country compared to western societies.

The use of chemotherapy to treat early stage breast cancer; both in the adjuvant and neoadjuvant settings, is widely practiced especially so among younger patients with high-risk pathological features. Chemotherapy, in this age group, may induce premature menopause with its associated poor fertility. Though fertility is considered one of the key aspects of quality of life for breast cancer survivors, it is usually poorly addressed at diagnosis, by both physicians and patients, alike! Given the nature of cancer as an illness, patients are usually overwhelmed with such diagnosis and their focus is usually on the cancer and how soon they will start active treatment. Many patients and families are usually under the impression that pursuing fertility preservation might delay their treatment, thus negatively affecting their cure rates. Additionally, patients may not be aware of their potential fertility loss, and such problem may not be communicated well by the treating physicians.^[15]^ A survey study of young female cancer survivors reported that feeling too distressed or overwhelmed at the time of diagnosis, lack of knowledge about fertility preservation options, and concerns about cost were the main barriers to fertility preservation.^[16]^

The issue of fertility might not be in the mind of many treating oncologist whose focus is chemotherapy and its immediate complications. A cross-sectional survey developed to self-evaluate knowledge, attitude, and behavior regarding fertility preservation of over 800 Japanese breast cancer specialists. Survey items included questions regarding knowledge and attitude toward fertility issues, fertility-related practice and potential barriers for the discussion of fertility with patients. Responding physicians also answered few questions related to their socio-demographic background. Among the 400 respondent physicians, female and younger oncologists (age less than 50 years) and those working in a multidisciplinary environment had significantly higher probability of referring patients to reproductive specialists. Physicians who scored better in knowledge items and positive attitudes toward fertility preservation were also more likely to discuss potential fertility issues with cancer patients. Higher risk of tumor recurrence, lack of collaborating reproductive specialists, and time constraints in the clinic were among the main barriers identified.^[17]^

In 2018, the American Society of Clinical Oncology issued an updated guideline recommending that health care providers discuss fertility preservation with all patients of child-bearing age planned to undergo anticancer treatment that may affect their fertility.^[18]^ Compliance with such guidelines continues to be a problem. In 1 retrospective study that included 303 women with breast cancer, aged 40 years or younger treated with chemotherapy and/or endocrine therapy, only 26% had fertility counseling with their physician; majority (89%) of them sought further fertility preservation options.^[19]^ Our counseling rates; approaching 90%, are significantly higher than previously reported. Our multidisciplinary approach to breast cancer care which mandates a full discussion with all patients diagnosed with early-stage breast cancer and having “fertility counseling” as a key performance indicator, in addition to having a “fertility-preservation” clinic had probably improved our compliance rates.

Definitions of amenorrhea is so variable in previously published studies and that may explain the wide range of reported POF and infertility rates especially after the use of taxanes. Some studies reported on rate of amenorrhea immediately after completion of chemotherapy^[20]^ while others reported rates at 3, 6, or 12 months.^[21]^ The impact of various definitions is illustrated by Padmanabhan et al, who reported the incidence of amenorrhea from the beginning of CMF chemotherapy at 3 months, 6 months, and 12 months later as 50%, 70%, and 80%, respectively.^[22]^

Our rates of amenorrhea, however, are relatively high. The sequential addition of taxanes to standard adjuvant anthracycline-based chemotherapy, resulted in statistically significant higher rates. The effect of duration of chemotherapy, in its own, is difficult to study in small studies like ours. Patients with longer duration of chemotherapy are those who were treated with taxanes thus extending the duration of chemotherapy. However, patients who had taxanes following anthracyclines for shorter duration (6 cycles) like the FEC-docetaxel regimen had lower rates of amenorrhea (45.7%) compared to those who had similar regimen but for 8 cycles; 69.2% with AC-paclitaxel and 66.7 with AC-docetaxel. Because the efficacy of many of the taxanes-containing chemotherapy regimens are almost equivalent, oncologist might consider giving the shorter regimen that is associated with less amenorrhea.

Patient-related factors, mainly age, may also contribute to the variable amenorrhea rates. In 1 study, Goldhirsch et al reported that 33% of women age younger than 40 years and 81% of women age older than 40 years become menopausal during adjuvant, classic CMF with oral cyclophosphamide for 6 months.^[23]^

The currently available options for fertility preservations and its associated cost are not encouraging, either. Ova or embryo cryopreservation may not be available within the same facility and lots of time, including our hospital, insurance coverage is not usually provided. In these situations, the use of GnRH agonists might be considered.^[24]^ Several clinical trials and meta-analyses have shown that premenopausal women who received ovarian function suppression using GnRH agonists while on chemotherapy were less likely to have ovarian failure and had higher rates of menses resumption compared to patients who did not. Obviously, this should not be viewed as alternative unless it is clear that patients might not be able to have the more standard ova cryopreservation done.

The adoption of multidisciplinary approach and comprehensive programs including “fertility-preservation” clinics in addition to special training and educational sessions for healthcare providers are necessary to improve on fertility counseling and meet the expectations of such young breast cancer patients.

## Author contributions

**Conception and design:** Hikmat Abdel-Razeq.

**Collection and assembly of data:** All authors.

**Data analysis and interpretation:** All authors.

**Manuscript writing:** All authors.

Final approval of manuscript: All authors.

Agree to be accountable for all aspects of the work: All authors.

Hikmat Abdel-Razeq orcid: 0000-0003-2833-6051.

## References

[1] Ghoncheh M Pournamdar Z Salehiniya H Incidence and mortality and epidemiology of breast cancer in the world. Asian Pac J Cancer Prev 2016;17 (S3):43–6. 10.7314/apjcp.2016.17.s3.43 27165206

[2] Bray F Ferlay J Soerjomataram I Global cancer statistics 2018: GLOBOCAN estimates of incidence and mortality worldwide for 36 cancers in 185 countries. CA Cancer J Clin 2018;68:394–424. 30207593 10.3322/caac.21492

[3] Khader YS Sharkas GF Arkoub KH The epidemiology and trend of cancer in Jordan 2000-2013. J Cancer Epidemiol 2018;2018:2937067. 30416523 10.1155/2018/2937067 PMC6207872

[4] Korde LA Partridge AH Esser M Breast cancer in young women: research priorities. A report of the young survival coalition research think tank meeting. J Adolesc Young Adult Oncol 2015;4:34–43. 26812429 10.1089/jayao.2014.0049

[5] Early Breast Cancer Trialists’ Collaborative Group. Polychemotherapy for early breast cancer: an overview of the randomised trials. Lancet 1998;352:930–42. 9752815

[6] Goh M Nguyen H Khan NN Identifying and addressing osteoporosis knowledge gaps in women with premature ovarian insufficiency and early menopause: a mixed-methods study. Clin Endocrinol (Oxf) 2019;91:498–507. 31218708 10.1111/cen.14049

[7] Buttros DAB Branco MT Orsatti CL High risk for cardiovascular disease in postmenopausal breast cancer survivors. Menopause 2019;26:1024–30. 31453965 10.1097/GME.0000000000001348

[8] Thurston RC Joffe H Vasomotor symptoms and menopause: findings from the Study of Women's Health across the Nation. Obstet Gynecol Clin North Am 2011;38:489–501. 21961716 10.1016/j.ogc.2011.05.006 PMC3185243

[9] Cohen LS SC Joffe H Diagnosis and management of mood disorders during the menopausal transition. Am J Med 2005;118: Suppl 12B: 93. 10.1016/j.amjmed.2005.09.042 16414333

[10] Goodwin PJ Ennis M Pritchard KI Risk of menopause during the first year after breast cancer diagnosis. J Clin Oncol 1999;17:2365–70. 10561298 10.1200/JCO.1999.17.8.2365

[11] Mamounas EP Bryant J Lembersky B Paclitaxel after doxorubicin plus cyclophosphamide as adjuvant chemotherapy for node-positive breast cancer: results from NSABP B-28. J Clin Oncol 2005;23:3686–96. 15897552 10.1200/JCO.2005.10.517

[12] Henderson IC Berry DA Demetri GD Improved outcomes from adding sequential paclitaxel but not from escalating doxorubicin dose in an adjuvant chemotherapy regimen for patients with node-positive primary breast cancer. J Clin Oncol 2003;315:976–98. 10.1200/JCO.2003.02.063 12637460

[13] Fornier MN Modi S Panageas KS Incidence of chemotherapy-induced, long-term amenorrhea in patients with breast carcinoma age 40 years and younger after adjuvant anthracycline and taxane. Cancer 2005;104:1575–9. 16134178 10.1002/cncr.21385

[14] Pursche T Bauer J Hammersen F Early-onset breast cancer: effect of diagnosis and therapy on fertility concerns, endocrine system, and sexuality of young mothers in Germany. Breast Care (Basel) 2019;14:23–9. 31019439 10.1159/000488795 PMC6465684

[15] Kelvin JF Thom B Benedict C Cancer and fertility program improves patient satisfaction with information received. J Clin Oncol 2016;34:1780–6. 27044937 10.1200/JCO.2015.64.5168 PMC4966338

[16] Benedict C Thom B Friedman DN Young adult female cancer survivors’ unmet information needs and reproductive concerns contribute to decisional conflict regarding post treatment fertility preservation. Cancer 2016;122:2101–9. 27213483 10.1002/cncr.29917 PMC4911318

[17] Shimizu C Bando H Kato T Physicians’ knowledge, attitude, and behavior regarding fertility issues for young breast cancer patients: a national survey for breast care specialists. Breast Cancer 2013;20:230–40. 22271066 10.1007/s12282-011-0328-8

[18] Oktay K Harvey BE Partridge AH Fertility preservation in patients with cancer: ASCO clinical practice guideline update. J Clin Oncol 2018;36:1994–2001. 29620997 10.1200/JCO.2018.78.1914

[19] McCray DK Simpson AB Flyckt R Fertility in women of reproductive age after breast cancer treatment: practice patterns and outcomes. Ann Surg Oncol 2016;23:3175–81. 27334218 10.1245/s10434-016-5308-y

[20] Berliere M Dalenc F Malingret N Incidence of reversible amenorrhea in women with breast cancer undergoing adjuvant anthracycline-based chemotherapy with or without docetaxel. BMC Cancer 2008;8:56. 18291033 10.1186/1471-2407-8-56 PMC2287183

[21] Tham YL Sexton K Weiss H The rates of chemotherapy-induced amenorrhea in patients treated with adjuvant doxorubicinand cyclophosphamide followed by a taxane. Am J Clin Oncol 2007;30:126–32. 17414460 10.1097/01.coc.0000251398.57630.4f

[22] Padmanabhan N Wang DY Moore JW Ovarian function and adjuvant chemotherapy for early breast cancer. Eur J Cancer Clin Oncol 1987;23:745–8. 3653192 10.1016/0277-5379(87)90272-0

[23] Goldhirsch A Gelber RD Castiglione M The magnitude of endocrine effects of adjuvant chemotherapy for premenopausal breast cancer patients: the International Breast Cancer Study Group. Ann Oncol 1990;1:183–8. 2261364 10.1093/oxfordjournals.annonc.a057718

[24] Abdel-Razeq H Gonadotropin-releasing hormone agonists during chemotherapy for ovarian function and fertility preservation for patients with early-stage breast cancer. Cancer Manag Res 2019;11:4273–82. 31190993 10.2147/CMAR.S204069 PMC6514123

